# Hit the gym or hit the hay: can evening exercise characteristics predict compromised sleep in healthy adults?

**DOI:** 10.3389/fphys.2023.1231835

**Published:** 2023-07-28

**Authors:** Dean J. Miller, Gregory D. Roach, Michele Lastella, Emily R. Capodilupo, Charli Sargent

**Affiliations:** ^1^ The Appleton Institute for Behavioural Science, Central Queensland University, Wayville, SA, Australia; ^2^ WHOOP Inc., Data Science and Research, Boston, MA, United States

**Keywords:** wearables, sleep quality, exercise participation, gradient boosting, body sensor networks, mobile health, exercise

## Abstract

**Introduction:** Recent sleep guidelines regarding evening exercise have shifted from a conservative (i.e., do not exercise in the evening) to a more nuanced approach (i.e., exercise may not be detrimental to sleep in circumstances). With the increasing popularity of wearable technology, information regarding exercise and sleep are readily available to the general public. There is potential for these data to aid sleep recommendations within and across different population cohorts. Therefore, the aim of this study was to examine if sleep, exercise, and individual characteristics can be used to predict whether evening exercise will compromise sleep.

**Methods:** Data regarding evening exercise and the subsequent night’s sleep were obtained from 5,250 participants (1,321F, 3,929M, aged 30.1 ± 5.2 yrs) using a wearable device (WHOOP 3.0). Data for females and males were analysed separately. The female and male datasets were both randomly split into subsets of training and testing data (training:testing = 75:25). Algorithms were trained to identify compromised sleep (i.e., sleep efficiency <90%) for females and males based on factors including the intensity, duration and timing of evening exercise.

**Results:** When subsequently evaluated using the independent testing datasets, the algorithms had sensitivity for compromised sleep of 87% for females and 90% for males, specificity of 29% for females and 20% for males, positive predictive value of 32% for females and 36% for males, and negative predictive value of 85% for females and 79% for males. If these results generalise, applying the current algorithms would allow females to exercise on ~ 25% of evenings with ~ 15% of those sleeps being compromised and allow males to exercise on ~ 17% of evenings with ~ 21% of those sleeps being compromised.

**Discussion:** The main finding of this study was that the models were able to predict a high percentage of nights with compromised sleep based on individual characteristics, exercise characteristics and habitual sleep characteristics. If the benefits of exercising in the evening outweigh the costs of compromising sleep on some of the nights when exercise is undertaken, then the application of the current algorithms could be considered a viable alternative to generalised sleep hygiene guidelines.

## Introduction

There is widespread consensus among the scientific community that exercise and sleep have a positive impact on human health (Hirshkowitz et al., 2015; Piercy and Troiano, 2018). Similarly, the negative impact of compromised sleep on human physiology and performance is well established (Belenky et al., 2003; Paryab et al., 2021; Khcharem et al., 2022). Despite this knowledge, the way in which exercise impacts sleep is less understood. Exercise has been promoted as a potential non-pharmacological intervention to improve sleep (Kovacevic et al., 2018; Si et al., 2020; Xie et al., 2021). However, a variable that may be disruptive to sleep is the time of day when exercise is completed. Historically, sleep medicine recommendations have taken a conservative approach, advising against exercise in close proximity to habitual bedtime (Zarcone, 1994; American Sleep Association, 2023). More recent recommendations are less conservative, with the Sleep Foundation stating that “most people” can perform moderate intensity exercise in the evening without impacting sleep, as long as there is a 90-min buffer between exercise and sleep (Pacheco, 2021). However, the only citation used for this recommendation is Miller et al. (2020b), a paper that investigated the impact of evening exercise only in healthy young males (Miller et al., 2020c). It is possible that individuals following this advice, who are not healthy young males, may be compromising their sleep by performing evening exercise. This highlights the difficulty and limitations of providing generalised recommendations regarding evening exercise and sleep.

Physiologically, the most likely mechanism for the impact of evening exercise on sleep is elevated core body temperature. During exercise, core body temperature is elevated as a by-product of muscular contraction. The human circadian rhythm of core body temperature coincides with sleep/wake behaviour, such that body temperature decreases as the body is primed for sleep, and body temperature is elevated during active hours (Van Dongen and Dinges, 2000). If exercise is performed close to habitual bedtime, the associated increase in body temperature could potentially disrupt the body’s physiological preparation for sleep (Waterhouse et al., 2005; Sawka et al., 2011; Miller et al., 2020c). In isolation of recommendations and potential mechanisms, the results of several laboratory and epidemiological studies indicate that evening exercise may not be detrimental to subsequent sleep in all circumstances (Myllymäki et al., 2011; Buman et al., 2014; Alley et al., 2015; Aloulou et al., 2020; Miller et al., 2020c; Thomas et al., 2020; Frimpong et al., 2021). While it is less common, some studies have reported negative effects of evening exercise on sleep, with a high intensity exercise protocol performed in the evening resulting in decreased sleep efficiency in elite male runners (Ramos-Campo et al., 2019). Contrasting findings surrounding this topic are likely due to different combinations of the characteristics of the exercise (i.e., modality, intensity, duration, timing). Depending on these characteristics, the physiological response to exercise can vary considerably (Myllymäki et al., 2012; Mann et al., 2014; Miller et al., 2020c). To control for such variability, research studies are often limited to one combination of exercise characteristics (i.e., type, intensity, duration) and sleep timing (i.e., bedtime and wake up time). This leads to contrasting outcomes across studies and practical applications that can only be applied to specific exercise protocols and to the sample demographic that participate.

Technology capable of non-invasively measuring sleep and other physiological markers of health over multiple days and nights provide an ideal framework in which to explore the efficacy of physiological markers for predicting health related outcomes (Miller et al., 2020a; Capodilupo and Miller, 2021; Miller et al., 2022). More specifically, datasets generated with the use of wearable technologies provide a wide array of exercise and demographic variables that can be used to examine the relationship between exercise and sleep (Capodilupo and Miller, 2021). Previous research has utilised daytime activity (i.e., movement) to effectively predict sleep quality (Sathyanarayana et al., 2016). However, no studies have utilised individual characteristics (e.g., age, fitness level), sleep characteristics (e.g., timing) and exercise characteristics (e.g., timing duration, heart rate) to predict sleep outcomes. Therefore, the aim of this study was to utilise data obtained from a wearable device (i.e., WHOOP 3.0) to predict the probability of compromised sleep based on metrics relating to sleep, and evening exercise.

## Methods

The WHOOP strap 3.0 (WHOOP Inc., Boston, United States) is a wearable device typically worn on the wrist. The device uses accelerometry to obtain actigraphy data (movement) and green and/or infrared LEDs paired with photodiodes to obtain photoplethysmography data (blood volume) to collect measures of sleep, heart rate, and other physiological markers of health (Miller et al., 2020b; Bellenger et al., 2021; Miller et al., 2021; Bellenger et al., 2022; Miller et al., 2022). Sleep and exercise periods are automatically detected by the device and transmitted via Bluetooth to associated Android and iOs smartphone applications for analysis. The specific algorithms used by WHOOP for estimating sleep and other physiological metrics are proprietary. The data used in this study were extracted from an existing database as part of a research collaboration between WHOOP Inc. and CQUniversity. The study was approved by the Central Queensland University Human Research Ethics Committee (Ethics number: 22344) in compliance with the Declaration of Helsinki. Data were collected with the written consent of individuals via WHOOP Inc.’s terms of service.

A subset of the existing dataset was extracted for the period between December 2019 and February 2020. For each day, the data were filtered for days that individuals 1) performed exercise after 19:00 h; 2) exercise duration was more than 30 min; and 3) had an average heart rate higher than 50% of maximum heart rate during exercise (HR) (Tanaka et al., 2001). A total of 5,250 individuals (1,321 females; 3,929 males; overall mean age ± SD = 30.1 ± 5.2; female mean age ±SD = 30.1 ± 5.0, male mean age ±SD = 29.9 ± 5.3) met the criteria and contributed 21,840 nights of data to the analyses. The following variables were obtained from the WHOOP platform:

• Sleep onset (hh: mm): time at which sleep started
• Sleep offset (hh: mm): time at which sleep ended
• Sleep period (h): time period between sleep onset and sleep offset
• Sleep efficiency (%): percentage of the sleep period spent asleep
• exercise end time (hh:mm): time of day at which an exercise ceased
• exercise intensity (% of maximum HR): average HR during exercise
• exercise duration (min): total duration of a bout of exercise
• Fitness level: hours of exercise per week

### Data analysis

Every observation in the master dataset was assigned a classification of either “uncompromised sleep” or “compromised sleep” based on sleep efficiency (i.e., >90% = uncompromised sleep; <90% = compromised; Table 1). The 90% threshold for compromised vs. uncompromised sleep was chosen as the sample demographic had high sleep efficiency (i.e., mean sleep efficiency ±SD = 90.1% ± 5.0%). If the current model is applied to a sample with lower sleep efficiency (e.g., mean efficiency <90%), the threshold should be lowered accordingly. The master dataset (n = 21,840) was split into female and male datasets to control for potential gender differences in sleep and exercise characteristics. Each of these datasets were then randomly split into training and testing datasets (75%/25% split). All individuals contributed to the training and testing datasets, with an average of 4.2 observations per participant. For the female cohort there were 2,707 uncompromised sleeps and 953 compromised sleeps in the training dataset, and there were 885 uncompromised sleeps and 335 compromised sleeps in the testing dataset. For the male cohort there were 8,547 uncompromised sleeps and 4,173 compromised sleeps in the training dataset, and there were 2,813 uncompromised sleeps and 1,427 compromised sleeps in the testing dataset. Due to the class imbalance between uncompromised and compromised sleeps in the both training datasets, synthetic samples of each predictor variable were generated for the positive class (i.e., compromised sleep) by adding uniformly distributed random noise to each compromised sleep, bringing the total of uncompromised sleeps to 1,906 for the female dataset and 8,346 for the male dataset (Siriseriwan, 2019).

**TABLE 1:** Agreement matrix for binary classification.

		Prediction	
		Compromised	Uncompromised
Truth Data	Compromised	True Compromised (TC)	False Uncompromised (FU)
	Uncompromised	False Compromised (FC)	True Uncompromised (TU)

A gradient boosted classifier (Kuhn, 2008) was trained to return a probability of an individual having compromised sleep (i.e., less than 90% sleep efficiency (Ohayon et al., 2004)) based on the following features:

• age
• fitness level
• average sleep midpoint (midpoint of habitual sleep start and habitual sleep end)
• exercise intensity
• exercise duration
• exercise end time
• habitual sleep start time

A gradient boosted classifier was chosen as it outperformed other learning models (e.g., logistic regression) and has been utilised in previous research examining similar datasets (Miller et al., 2020a). The model provides a prediction statistic between 0 and 1, with a higher value indicating higher confidence in the prediction. To provide a binary classification, a threshold at which the prediction statistic classifies an observation in the positive class (i.e., compromised sleep), or negative (i.e., uncompromised sleep) must be established. The models for both female and male cohorts were run with thresholds ranging from 0.05 to 0.95 in 0.05 intervals (Table 2). Thresholds that provided high prediction for compromised sleep (i.e., >85% sensitivity) and reasonable prediction for uncompromised sleep (i.e., >20% specificity) were chosen as the adjusted thresholds. This was done to maximise the sensitivity of the model for the “low-risk” outcomes (i.e., compromising sleep by incorrectly recommending exercise). Therefore, two thresholds; 1) a software default threshold of 0.5 (Kuhn, 2008) (i.e., prediction statistic >0.5 = compromised sleep); and 2) an adjusted threshold optimised to provide to predict compromised sleep (females = 0.3, males = 0.4; i.e., prediction statistic>adjusted threshold = compromised sleep) were used in this analysis.

**TABLE 2:** Model performance with adjusted threshold values.

Threshold	Sensitivity	Specificity	PPV	NPV
*Females*				
0.05	100	0	27	NA
0.10	99	0	28	83
0.15	99	0	28	84
0.20	97	1	28	87
0.25	94	15	30	87
0.30	87	29	32	85
0.35	75	49	35	83
0.40	60	67	40	81
0.45	43	81	46	79
0.50	30	92	57	77
0.55	27	97	76	79
0.60	20	97	71	76
0.65	11	99	87	75
0.70	6	99	90	74
0.75	0	100	100	73
0.80	0	100	100	73
0.85	0	100	100	73
0.90	0	100	NA	73
0.95	0	100	NA	73
*Males*				
0.05	100	0	33	NA
0.10	100	0	33	NA
0.15	99	0	34	80
0.20	99	0	34	94
0.25	99	0	34	91
0.30	99	0	34	86
0.35	97	1	35	82
0.40	90	20	36	79
0.45	70	41	38	73
0.50	44	70	43	71
0.55	19	91	52	69
0.60	6	98	59	67
0.65	3	99	79	67
0.70	2	99	86	67
0.75	1	99	90	67
0.80	0	99	85	67
0.85	0	99	83	66
0.90	0	100	NA	66
0.95	0	100	NA	66

To assess the predictive ability of the models using both thresholds, the following statistics were calculated:

• sensitivity (%) = TC/(TC + FU)*100—i.e., percentage of compromised sleeps correctly predicted by the model
• specificity (%) = TU/(TU + FC)*100—i.e., percentage of uncompromised sleep correctly predicted by the model
• positive predictive value (%) = TC/(TC + FC)*100—i.e., percentage of sleeps predicted to be compromised that were compromised
• negative predictive value (%) = TU/(TU + FU)*100—i.e., percentage of sleep predicted to be uncompromised that were uncompromised
• feature importance: ranking of the importance of each feature within the model (Kuhn, 2008)

## Results

**FIGURE 1:**
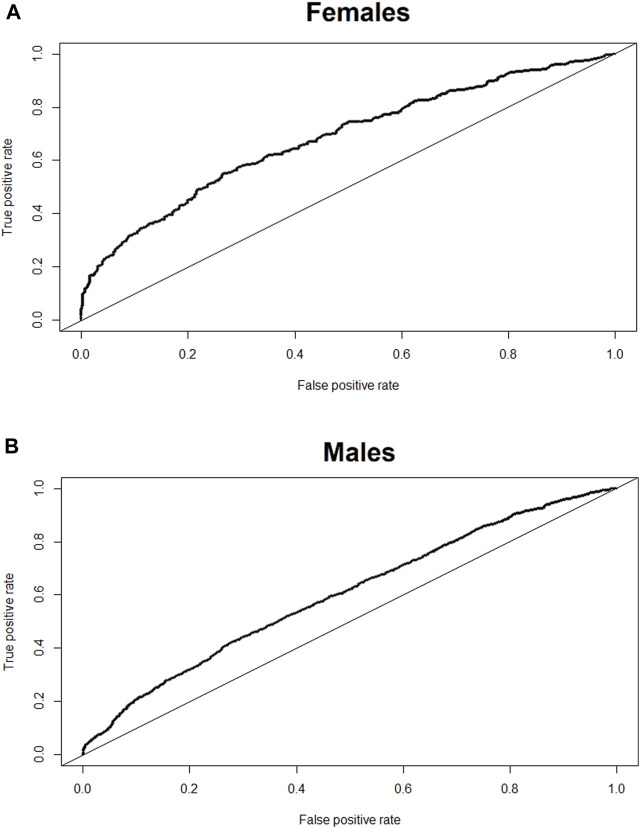
Receiver operating characteristic curve for each model. Females **(A)**, and males **(B)**.

**FIGURE 2:**
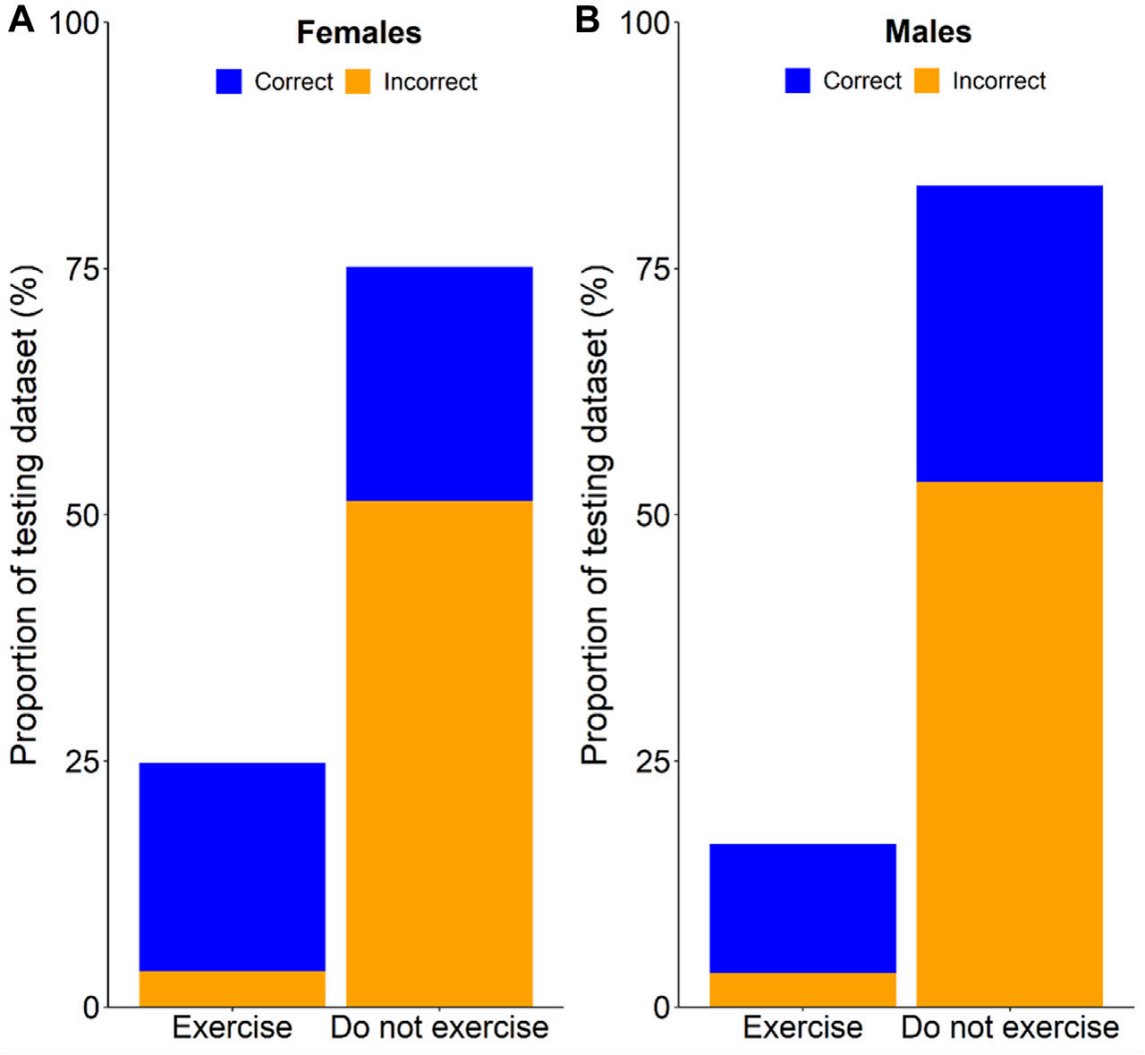
Cumulative percentage of correct and incorrect exercise recommendations for females **(A)**, and males **(B)**.

The models returned a continuous probability that predictor variables are indicative of reduced sleep efficiency (i.e., <90). Receiver operator curves (Figure 1) and prediction statistics (Table 2) summarise the performance of the models after mapping the model’s continuous probability output into binary classifications, bifurcated on thresholds ranging from 0.05 to 0.95 in 0.05 intervals. An adjusted threshold was chosen for females and males to maximise the sensitivity of the model (i.e., correctly predicting compromised sleep) without compromising the specificity of the model (i.e., correctly classifying uncompromised sleep; Tables 3, 4) (Miller et al., 2020a). Figure 2 demonstrates how the model may be used practically, with model predictions (i.e., compromised sleep or uncompromised sleep) assigned an exercise recommendation based on the binary classification. A recommendation of “exercise” is assigned if the model predicts uncompromised sleep and “do not exercise” is assigned when the model predicts compromised sleep.

**TABLE 3:** Model performance for the classification of compromised and uncompromised sleeps in the female cohort (default and chosen adjusted threshold).

Dataset	Sensitivity (%)	Specificity (%)	PPV (%)	NPV (%)
*Training dataset (raw)*				
Default threshold (0.5)	37	92	63	81
Adjusted threshold (0.3)	92	31	32	92
*Training dataset (+synthetic data)*				
Default threshold (0.5)	47	92	81	71
Adjusted threshold (0.3)	95	31	49	90
*Testing dataset*				
Default threshold (0.5)	30	92	57	77
Adjusted threshold (0.3)	87	29	32	85

Note: PPV, positive predictive value; NPV, negative predictive value.

**TABLE 4:** Model performance for the classification of compromised and uncompromised sleeps in the male cohort (default and chosen adjusted threshold).

Dataset	Sensitivity (%)	Specificity (%)	PPV (%)	NPV (%)
*Training dataset (raw)*				
Default threshold (0.5)	48	73	47	74
Adjusted threshold (0.4)	91	21	36	82
*Training dataset (+synthetic data)*				
Default threshold (0.5)	58	73	68	64
Adjusted threshold (0.4)	94	21	54	77
*Testing dataset*				
Default threshold (0.5)	44	70	43	71
Adjusted threshold (0.4)	90	20	36	79

Note: PPV, positive predictive value; NPV, negative predictive value.

### Females

**FIGURE 3:**
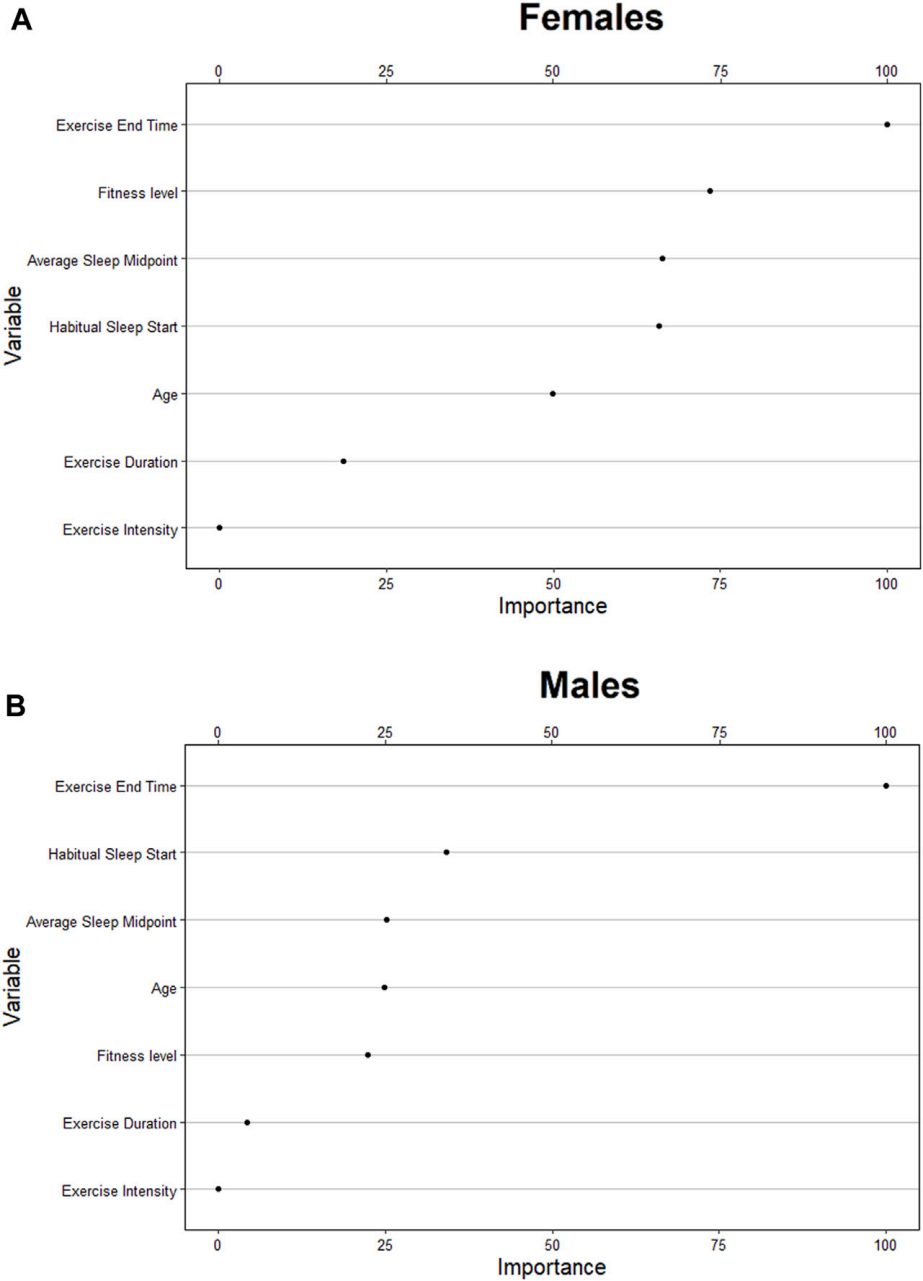
Relative ranking of the feature importance for individual (age, fitness level), sleep (habitual start, midpoint, exercise-to-sleep period) and exercise characteristics (duration, intensity) for females **(A)**, and males **(B)**.

On average, the female cohort fell asleep at 21:45 ± 1.92 h, obtained 7.67 ± 2.86 h of sleep, exercised at 68.04% ± 8.70% of maximum HR for 59.49 ± 40.94 min, and ceased exercise at 20:34 ± 1.69 h. The predictive model correctly classified 30% of compromised sleeps (i.e., sensitivity) when bifurcated by the default threshold, and correctly classified 87% of compromised sleeps (i.e., sensitivity) when bifurcated by the adjusted threshold (Table 3). The model correctly classified 92% of uncompromised sleeps (i.e., specificity) when bifurcated by the default threshold (i.e., 0.5), and correctly classified 29% of uncompromised sleeps (i.e., specificity) when bifurcated by the adjusted threshold (Table 3). The model correctly predicted 57% of sleeps that were actually compromised (i.e., PPV) when bifurcated by the default threshold (Table 3), and correctly predicted 32% of sleeps that were actually compromised (i.e., PPV) when bifurcated by the adjusted threshold (Table 3). The model correctly predicted 77% of sleeps that were actually uncompromised (i.e., NPV) when bifurcated by the default threshold (Table 3), and correctly predicted 85% of sleeps that were actually uncompromised (i.e., NPV) when bifurcated by the adjusted threshold (Table 3). Analysis of feature importance shows that workout end time, fitness level, average sleep midpoint, and habitual sleep start were the most powerful predictors in this model (Figure 3).

### Males

On average, the male cohort slept at 21:45 ± 1.92h, obtained 7.34 ± 4.60 h of sleep, exercised at 67.68% ± 9.10% of maximum HR for 59.31 ± 43.96 min, and ceased exercise at 20:53 ± 1.62 h. The predictive model correctly classified 44% of compromised sleeps (i.e., sensitivity) when bifurcated by the default threshold, and correctly classified 90% of compromised sleeps (i.e., sensitivity) when bifurcated by the adjusted threshold (Table 4). The model correctly classified 70% of uncompromised sleeps (i.e., specificity) when bifurcated by the default threshold (i.e., 0.5), and correctly classified 20% of uncompromised sleeps (i.e., specificity) when bifurcated by the adjusted threshold (Table 4). The model correctly predicted 43% of sleeps that were actually compromised (i.e., PPV) when bifurcated by the default threshold (Table 4), and correctly predicted 36% of sleeps that were actually compromised (i.e., PPV) when bifurcated by the adjusted threshold (Table 4). The model correctly predicted 71% of sleeps that were actually uncompromised (i.e., NPV) when bifurcated by the default threshold (Table 4), and correctly predicted 79% of sleeps that were actually uncompromised (i.e., NPV) when bifurcated by the adjusted threshold (Table 4). Analysis of feature importance shows that workout end time, habitual sleep start, and average sleep midpoint were the most powerful predictors in this model (Figure 3).

## Discussion

The present study utilised data obtained from the WHOOP strap 3.0 to provide insights into the relationship between evening exercise and subsequent sleep. The main finding was that the predictive model correctly classified 87% of compromised sleeps for females, and 90% of compromised sleeps for males when bifurcated by an adjusted threshold. Along with this high sensitivity, the high negative predictive value (i.e., percentage of uncompromised sleep correctly categorised) for females and males suggests that an individual can be confident that sleep will not be compromised if these models provide that recommendation.

The rationale behind the adjusted thresholds in the current study was to maximise the sensitivity of the model for the “low-risk” outcomes. The sole high-risk outcome for these models was the misclassification of a compromised sleep as an uncompromised sleep. If a model prediction of uncompromised sleep is assigned the recommendation of “exercise”, an individual may exercise in the evening and be confident that sleep will not be compromised. If the prediction is that sleep will be compromised with a recommendation of “do not exercise”, there will be a high percentage of times when exercise could have been undertaken but was not. While this is the main weakness of the model, when compared to avoidance of evening exercise in all situations, the current models would (1) provide the benefit of exercising on 25% of nights, but with only 15% of those nights being compromised for females (Figure 2), and (2) provide the benefit of exercising on 17% of nights, but with only 21% of those nights being compromised for males (Figure 2). If an individual considers the ability to be active on nights when they otherwise would not (25% for females; 17% for males) as more beneficial than the cost of having sleep compromised on a small percentage of those nights (15% for females; 21% for males), then the current models can be considered a viable alternative to generalised sleep hygiene recommendations. Furthermore, if the models are contextualised to an individual who would ordinarily exercise in the evening each night, following the current models would save them from compromising their sleep on 24% of nights (females) and 30% of nights (males; Figure 2).

The aim of these models is not to recommend evening exercise *in lieu* of morning or daytime exercise, but to provide evening exercise as an alternative for individuals who may not have the opportunity to otherwise be active. This is an important outcome given that lack of time is commonly identified as a barrier to undertaking daily exercise (Cerin et al., 2010). Individuals with a small window of “free time” may have to choose between prioritising exercise or sleep (Miller et al., 2020c). However, if models like the one presented in this study can be applied to easily accessible continuous wearable data, individuals may be able to consider evening exercise as a viable alternative to morning or afternoon exercise.

The predictors related to sleep and exercise utilised in this study are standard metrics, available to anyone with a validated wearable device. It is likely that wearable technology companies may be able to utilise proprietary algorithms and individualised data to create algorithms with better performance than the model presented in this study. For example, the definition of compromised sleep, while similar to previous research (Sathyanarayana et al., 2016), may be considered generalised rather than individualised. That is, the 90% sleep efficiency “cut-off” may be less sensitive for some individuals. If an individual that averages 97% sleep efficiency experiences a reduction to 91%, it would not be classified as “compromised” by the present model. While a sleep efficiency of 91% would not be considered clinically low sleep efficiency, it would translate to ∼29 min less sleep for a typical 8-h sleep and is therefore relevant for the individual. It is also important to consider the methodology used to calculate sleep efficiency with the WHOOP device. Sleep efficiency thresholds for sleep research are often applied to sleep measurement systems that acquire manual initiation of “time in bed” period, which form the basis of sleep efficiency calculations in that context (i.e., sleep efficiency = total sleep/time in bed * 100) (Sathyanarayana et al., 2016). The WHOOP device, along with most modern wearables, automatically detect sleep with no input from the user. Devices operating under this functionality detect the initiation of sleep rather than the start of time in bed (i.e.,. sleep efficiency = total sleep/sleep period * 100). It is possible that this methodology in combination with the healthy sample resulted in a higher sleep efficiency compared to previous studies. Which is another reason why a higher threshold for the classification of compromised sleep was used for this study.

The analysis of feature importance shows that features such as habitual sleep start, fitness level, and average sleep midpoint were strong predictors in the models (Figure 3). This suggests that variables relating to an individual’s habitual behaviour may be strong predictors of sleep quality in conjunction with acute exercise variables, supporting the notion that algorithms tailored to individual behaviours may outperform the current model. A limitation of the current model includes the lack of experimental control for other behavioural factors (e.g.,. meal size and/or timing), environmental factors (e.g., indoor/outdoor exercise/season of the year), and additional individual characteristics (e.g., body mass index, menstruation). Wearable technology and associated software are now able to collect self-reported behavioural data, which could be used as a predictor in future models.

Another way in which such algorithms could be improved is by including additional variables that are not readily available, or easily interpretable within smartphone applications. The emergence of temperature sensors and associated data could prove to be a valuable predictor of disturbed sleep. One of the main concerns for evening exercise is that core body temperature increases as a function of muscular contraction (Waterhouse et al., 2005; Sawka et al., 2011; Miller et al., 2020c)—which may impact the onset of sleep (Ramos-Campo et al., 2019). However, a laboratory study investigating this relationship has shown that moderate evening exercise did not result in elevated body temperature at bedtime following a 90-min exercise sleep latency and did not impact sleep (Miller et al., 2020c). If accurate temperature data can be obtained, a metric related to the reduction of core body temperature during the exercise-to-sleep period could be a powerful predictor of sleep quality.

## Conclusion

The main finding of this study was that the models were able to predict a high percentage of nights with compromised sleep based on individual characteristics, exercise characteristics and habitual sleep characteristics. Therefore, individuals may be confident that their sleep will not be compromised if the models recommend that exercise will not impact their sleep. Compared to avoidance of evening exercise (i.e., do not exercise in the evening), the current models allow for some evening exercise to be performed with only a small percentage of sleeps being compromised. Interpretations of the results should be made with boundary conditions in mind. Participants included in the study were physically active; it is unclear whether similar results would be obtained with less active individuals. Future investigations could assess individual-level algorithms to provide daily recommendations regarding the timing, type, intensity, and duration of exercise that can be without compromising sleep.

## Data Availability

The datasets presented in this article are not readily available because Data is owned by WHOOP Inc. and is restricted due to privacy policies. Requests to access the datasets should be directed to d.j.miller@cqu.edu.au.
